# Stereoselective Alkane Oxidation with *meta*-Chloroperoxybenzoic Acid (MCPBA) Catalyzed by Organometallic Cobalt Complexes †

**DOI:** 10.3390/molecules21111593

**Published:** 2016-11-22

**Authors:** Georgiy B. Shul’pin, Dmitriy A. Loginov, Lidia S. Shul’pina, Nikolay S. Ikonnikov, Vladislav O. Idrisov, Mikhail M. Vinogradov, Sergey N. Osipov, Yulia V. Nelyubina, Polina M. Tyubaeva

**Affiliations:** 1 Semenov Institute of Chemical Physics, Russian Academy of Sciences, ulitsa Kosygina, dom 4, Moscow 119991, Russia; 2 Chair of Chemistry and Physics, Plekhanov Russian University of Economics, Stremyannyi pereulok, dom 36, Moscow 117997, Russia; popov.AnA@rea.ru; 3 Nesmeyanov Institute of Organoelement Compounds, Russian Academy of Sciences, ulitsaVavilova, dom 28, Moscow 119991, Russia; dloginov@ineos.ac.ru (D.A.L.); shulpina@ineos.ac.ru (L.S.S.); ikonns@ineos.ac.ru (N.S.I.); vladchem32@gmail.com (V.O.I.); mmvngrdv@gmail.com (M.M.V.); osipov@ineos.ac.ru (S.N.O.); unelya@xrlab.ineos.ac.ru (Y.V.N.)

**Keywords:** alkanes, alcohols, metal complexes, X-ray analysis, *meta*-chloroperoxybenzoic acid (MCPBA), stereoselective oxidation

## Abstract

Cobalt pi-complexes, previously described in the literature and specially synthesized and characterized in this work, were used as catalysts in homogeneous oxidation of organic compounds with peroxides. These complexes contain pi-butadienyl and pi-cyclopentadienyl ligands: [(tetramethylcyclobutadiene)(benzene)cobalt] hexafluorophosphate, [(C_4_Me_4_)Co(C_6_H_6_)]PF_6_ (**1**); diiodo(carbonyl)(pentamethylcyclopentadienyl)cobalt, Cp*Co(CO)I_2_ (**2**); diiodo(carbonyl)(cyclopentadienyl)cobalt, CpCo(CO)I_2_ (**3**); (tetramethylcyclobutadiene)(dicarbonyl)(iodo)cobalt, (C_4_Me_4_)Co(CO)_2_I (**4**); [(tetramethylcyclobutadiene)(acetonitrile)(2,2′-bipyridyl)cobalt] hexafluorophosphate, [(C_4_Me_4_)Co(bipy)(MeCN)]PF_6_ (**5**); bis[dicarbonyl(B-cyclohexylborole)]cobalt, [(C_4_H_4_BCy)Co(CO)_2_]_2_ (**6**); [(pentamethylcyclopentadienyl)(iodo)(1,10-phenanthroline)cobalt] hexafluorophosphate, [Cp*Co(phen)I]PF_6_ (**7**); diiodo(cyclopentadienyl)cobalt, [CpCoI_2_]_2_ (**8**); [(cyclopentadienyl)(iodo)(2,2′-bipyridyl)cobalt] hexafluorophosphate, [CpCo(bipy)I]PF_6_ (**9**); and [(pentamethylcyclopentadienyl)(iodo)(2,2′-bipyridyl)cobalt] hexafluorophosphate, [Cp*Co(bipy)I]PF_6_ (**10**). Complexes **1** and **2** catalyze very efficient and stereoselective oxygenation of tertiary C–H bonds in isomeric dimethylcyclohexanes with MCBA: cyclohexanols are produced in 39 and 53% yields and with the *trans*/*cis* ratio (of isomers with mutual *trans*- or *cis*-configuration of two methyl groups) 0.05 and 0.06, respectively. Addition of nitric acid as co-catalyst dramatically enhances both the yield of oxygenates and stereoselectivity parameter. In contrast to compounds **1** and **2**, complexes **9** and **10** turned out to be very poor catalysts (the yields of oxygenates in the reaction with *cis*-1,2-dimethylcyclohexane were only 5%–7% and *trans*/*cis* ratio 0.8 indicated that the oxidation is not stereoselective). The chromatograms of the reaction mixture obtained before and after reduction with PPh_3_ are very similar, which testifies that alkyl hydroperoxides are not formed in this oxidation. It can be thus concluded that the interaction of the alkanes with MCPBA occurs without the formation of free radicals. The complexes catalyze oxidation of alcohols with *tert*-butylhydroperoxide (TBHP). For example, *tert*-BuOOH efficiently oxidizes 1-phenylethanol to acetophenone in 98% yield if compound **1** is used as a catalyst.

## 1. Introduction

Selective functionalization of C–H bonds in hydrocarbons and other compounds is a very real aim of contemporary catalytic science [1,2,3,4]. Numerous transition metal complexes play roles of efficient catalysts in oxygenation of hydrocarbons as well as alcohols [5,6,7,8,9,10,11,12,13,14]. Derivatives of cobalt are known to catalyze various transformations of organic compounds [15,16,17,18,19,20,21,22,23,24]. Molecular oxygen, hydrogen peroxide, *tert*-butyl hydroperoxides, and peroxyacids were used as oxidants in these oxygenations. Certain complexes of transition metals have been previously reported to oxidize organic compounds including alkanes with *meta*-chloroperoxybenzoicacid (MCPBA [25]) [25,26,27,28,29,30,31,32,33,34,35,36,37,38,39,40,41,42,43,44] (see, for example, complexes of cobalt [21,24,38], manganese [26,27,34,36,44], iron [28], nickel [30,31,32,33,35,37,39,42,43] and vanadium [40]). Earlier, Nam [15,16] reported that alkane oxidation with MCPBA catalyzed by some cobalt compounds proceeds stereoselectively. It is interesting to check the oxidation various cobalt organometallic complexes bearing ligands of different types, π-cyclobutadienyl, cyclopentadienyl, π-arene, carbonyl, amine, etc. derivatives, and compare both their activity in the reaction and stereoselectivity. It is also attractive to find any dependence of the product yield and stereoselectivity parameter on the structure of the particular complex. Complexes containing various ligands in different compositions have been chosen.

In the present work, we studied oxidation of alkanes and alcohols with peroxides catalyzed by certain organometallic derivatives of cobalt. Many of the complexes have been synthesized and characterized in this work for the first time.

## 2. Results and Discussion

### 2.1. Catalysts, Substrates and Oxidants

**Scheme 1:**
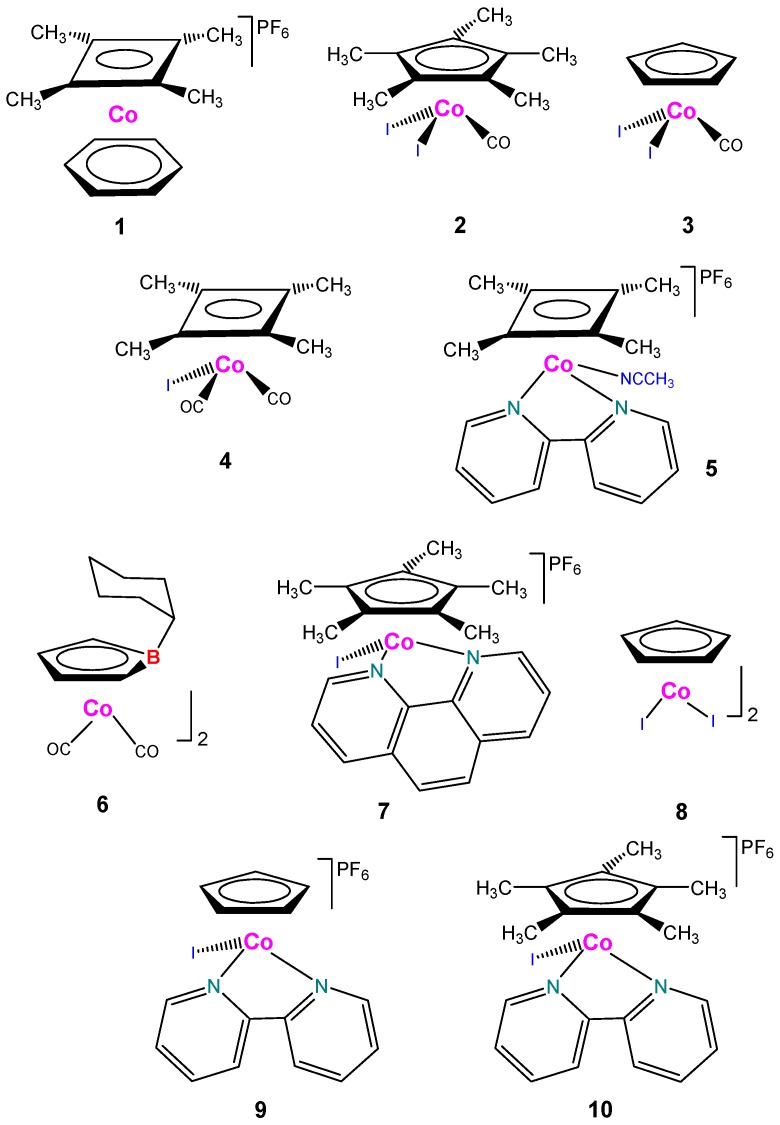
Structural formulae of the catalysts used in this work.

We used various cyclobutadienyl and cyclopentadienyl derivatives of cobalt as catalysts for oxidation of organic compounds with peroxides. Structural formulae of the catalysts are depicted in Scheme 1.

### 2.2. Syntheses of Catalysts

Syntheses of new complexes obtained in this work are described in Section 3.2. Complex [CpCo(bipy)I]PF_6_ (**9**) was prepared in high yield by the described in the literature method but with the usage of the improved counterion-exchange procedure (see Section 3.2). The related pentamethylated complexes with N,N-ligands, [Cp*Co(phen)I]PF_6_ (**7**) and [Cp*Co(bipy)I]PF_6_ (**10**), were synthesized in similar way by reactions of Cp*Co(CO)I_2_ (**2**) with 1,10-phenanthroline and 2,2′-bipyridyl. The cyclobutadiene complex [(C_4_Me_4_)Co(bipy)(MeCN)]PF_6_ (**5**) was prepared by photochemical replacement of benzene ligand in [(C_4_Me_4_)Co(C_6_H_6_)]PF_6_ (**1**) by 2,2′-bipyridyl in acetonitrile solution. All cationic complexes described here were isolated as salts with the PF_6_^−^ anion. All catalysts are indefinitely stable in air in solid state.

### 2.3. Structures of Catalysts

**Figure 1:**
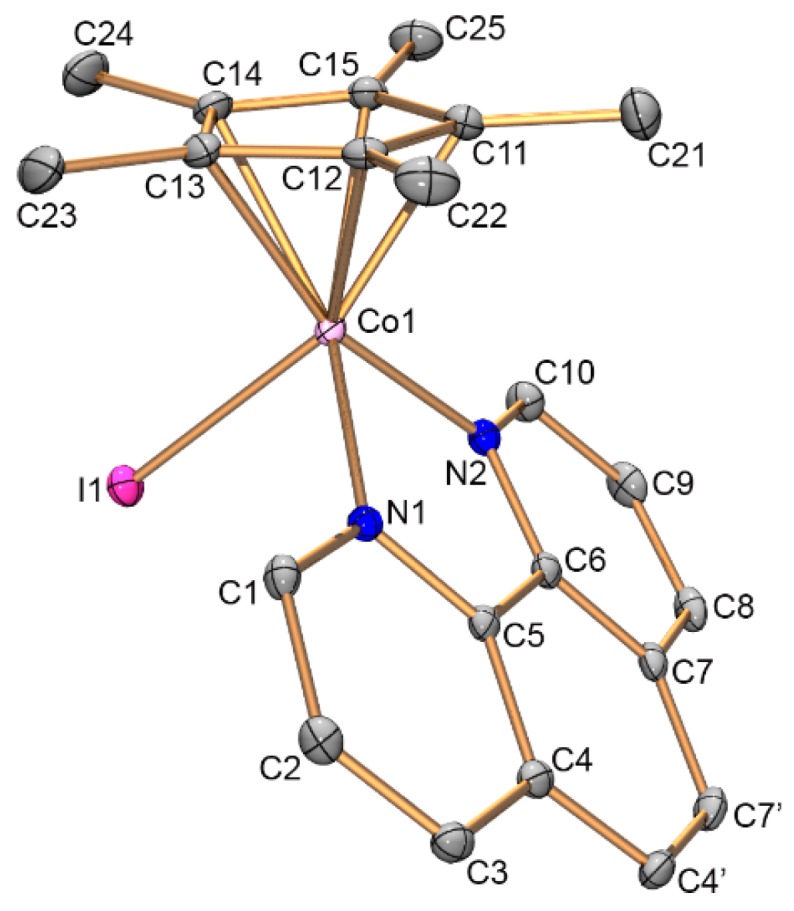
Structure of cation [Cp*Co(phen)I]^−^ (**7**). Atoms are represented by 50% thermal ellipsoids. Hydrogen atoms are omitted for clarity. Selected bond lengths [Å] and angles [°]: Co1—C11 2.059(3), Co1—C12 2.079(3), Co1—C13 2.061(3), Co1—C14 2.099(3), Co1—C15 2.097(3), Co1—N1 1.958(2), Co1—N2 1.968(2), Co1—I1 2.5798(4), and N1—Co1—N2 82.85(9).

**Figure 2:**
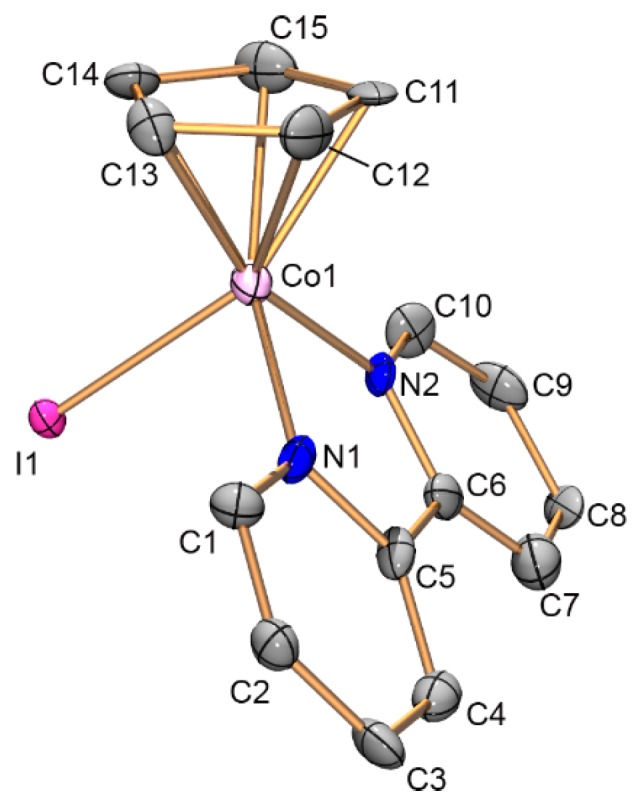
Structure of cation [CpCo(bipy)I]^−^ (**9**). Atoms are represented by 50% thermal ellipsoids. Hydrogen atoms are omitted for clarity. Selected bond lengths [Å] and angles [°] for the first/second symmetry-independent species: Co1—C11 2.046(17)/2.049(17), Co1—C12 2.06(2)/2.050(16), Co1—C13 2.056(17)/2.063(17), Co1—C14 2.127(17)/2.061(16), Co1—C15 2.072(19)/2.045(17), Co1—N1 1.932(14)/1.936(15), Co1—N2 1.966(13)/1.956(17), Co1—I1 2.574(3)/2.575(2), and N1—Co1—N2 82.2(5)/82.7(6).

**Figure 3:**
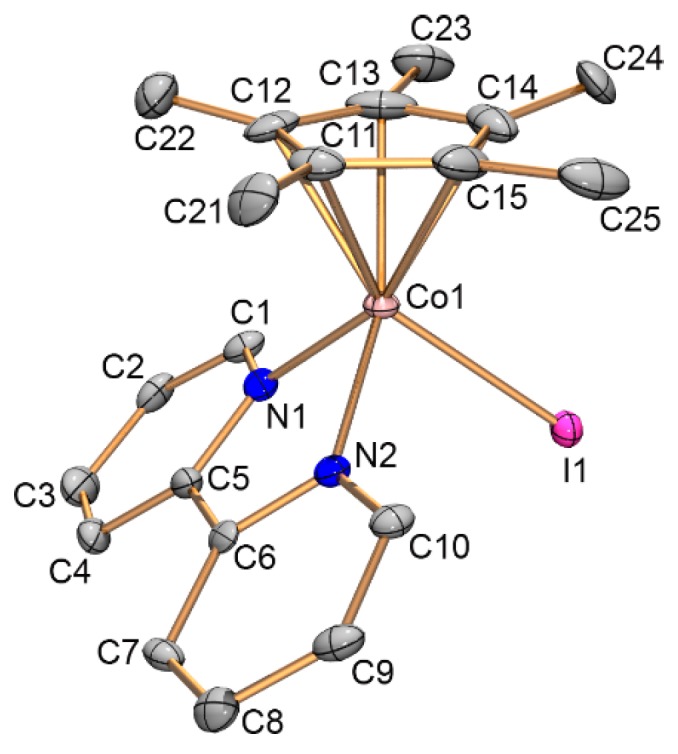
Structure of cation [Cp*Co(bipy)I]^−^ (**10**). Atoms are represented by 50% thermal ellipsoids. Hydrogen atoms are omitted for clarity. Selected bond lengths [Å] and angles [°]: Co1—C11 2.048(10), Co1—C12 2.066(10), Co1—C13 2.074(12), Co1—C14 2.095(10), Co1—C15 2.065(11), Co1—N1 1.965(8), Co1—N2 1.954(8), Co1—I1 2.5718(13), and N1—Co1—N2 81.6(3).

Complexes **7**, **9**, and **10** were investigated by X-ray diffraction. Their molecular structures, bond lengths and angles as well as crystallographic data and structure refinement parameters are given in Figure 1, Figure 2 and Figure 3 as well as in Section 3.3.

In the case of **9**, the symmetry-independent part of the unit cell contains two formula units. All complexes have a piano-stool geometry. The bi-pyridine ligand in **9** and **10** is almost planar; the dihedral angle formed by the intersection of the planes defined by the pyridyl rings is equal to 5.83 (6.55) and 8.78°, respectively. The Co···C_5_ distance in pentamethylated complexes **10** (1.691 Å) and **7** (1.687 Å) is somewhat longer than the corresponding distance in the unsubstituted derivative **1** (1.667 and 1.683 Å, average 1.675 Å); this can be explained by sterical effect of five methyl groups. The length of the Co–I bond in the three complexes **7**, **9** and **10** varies in the range 2.5718(13)–2.5798(4) Å.

The main geometric parameters of **10** resembles those in a recently characterized chloride analog [Cp*Co(bipy)Cl]PF_6_ [45]. For example, the dihedral angle between the Cp and bipy planes in **10** (38.2°) is close to that in [Cp*Co(bipy)Cl]PF_6_ (41.5°) but smaller than in **9** (average 50.1°) and **7** (49.2°).

### 2.4. Determination of Alkyl Hydroperoxides by GC before and after Reduction with PPh_3_

Earlier, one of us developed a convenient method for the analysis of mixtures obtained in alkane oxidation with molecular oxygen or peroxides [46,47,48,49,50,51,52,53,54,55] (the Shul’pin method [56]). In many cases, oxidation leads to the formation of three main products (alkyl hydroperoxide, alcohol and ketone). If a reaction solution is injected *directly* (see, for example, [57,58,59,60,61,62]) to the chromatograph, the alkyl hydroperoxide ROOH (if present) decomposes in the injector and/or column to produce the alcohol and ketone in approximately equal amounts.

However, if a sample of the reaction mixture is reduced by an excess of solid PPh_3_ (or thiourea) during 10–20 min, the alkyl hydroperoxide is quantitatively transformed into the corresponding alcohol. Comparing concentrations of the alcohol and ketone before and after treatment with PPh_3_, we can qualitatively conclude on existence or non-existence of ROOH in the solution [63,64,65,66,67,68,69,70,71]. Moreover, the *real* concentrations of the alkyl hydroperoxide as well as of alcohol and ketone can be calculated (estimated) using the data obtained *before* and *after* reduction [72,73,74,75,76,77,78,79,80,81]. Selectivity of the oxidation reaction can be characterized by the parameter [ROOH + A]/[K] where [ROOH + A] and [K] are concentrations of alcohol and ketone after reduction with PPh_3_, respectively [82,83]. However, this parameter gives us absolutely no information on existence or non-existence of ROOH in the solution.

We must emphasize that, although treatment with PPh_3_ can be used in order to *only* remove from the reaction mixtures peroxides (starting H_2_O_2_, *tert*-BuOOH, produced ROOH etc.) [84,85,86,87,88,89,90,91], the main aim of the method under discussion is estimation of real concentrations of ROOH, alcohol and ketone formed in the reaction. Chromatograms obtained *only* after reduction of the reaction mixture cannot be used for determination of *real* concentration of each oxygenate. Nevertheless, such chromatograms give valuable information on the total concentration of produced oxygenates.

### 2.5. Oxidation of Alkanes and Alcohols with Peroxides

**Scheme 2:**
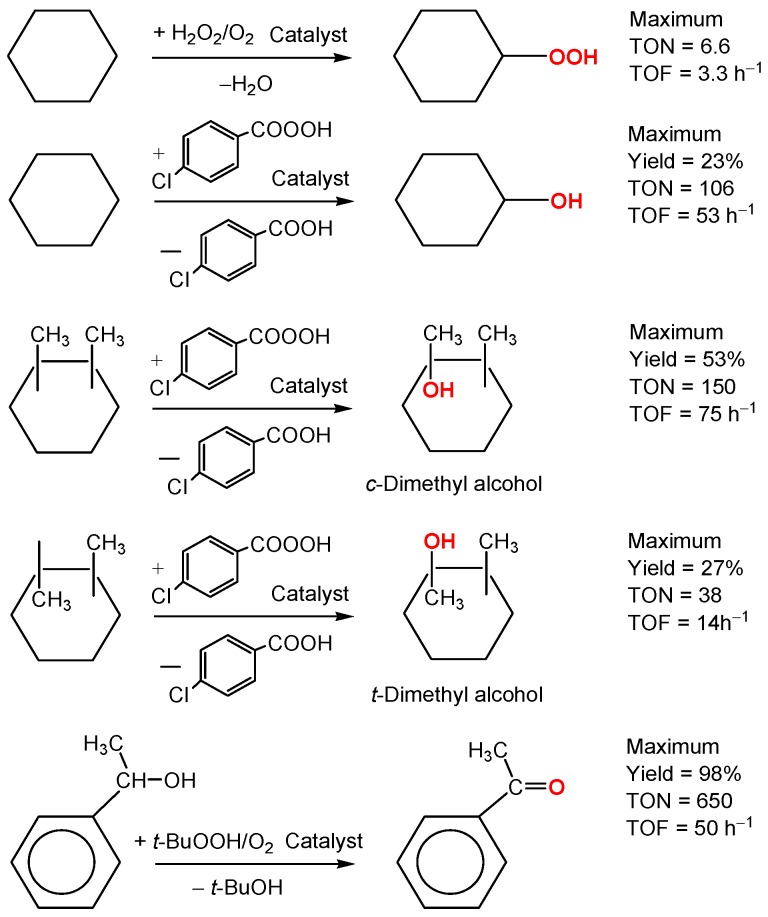
Examples of oxidation reactions studied in this work.

We have tested the catalytic effect of compound **1**–**10** in the reactions of alkanes and alcohols with various oxidants. These reactions are summarized in Scheme 2.

All complexes exhibited low activity in the oxidations with hydrogen peroxide. Some examples are presented in Table 1. The oxidation affords predominantly cyclohexyl hydroperoxide because the treatment of the reaction solution with PPh_3_ leads to the decrease of the ketone peak and increase of the alcohol peak in GC [55] (compare entries 1 and 2 in Table 1).

**Figure 4:**
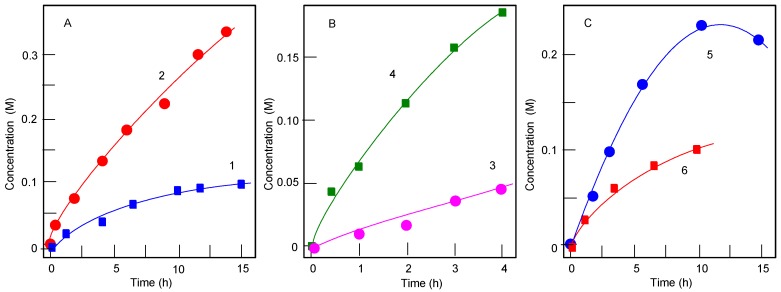
The 1-phenylethanol (0.33 M) oxidation with *tert*-butyl hydroperoxide (TBHP; 70%, aqueous, 1.65 M): (**A**) Accumulation of acetophenone with time in in the absence (curve 1) and in the presence (curve 2) of compound **1** (5 × 10^−4^ M). The reaction gave TON 650 and TOF 50 h^‒1^. Temperature was 70 °C, solvent was acetonitrile (total volume of the reaction solution was 5 mL); (**B**) The same oxidation catalyzed by complex **4** at 50 (curve 3) and 70 °C (curve 4); (**C**) The same oxidation ([TBHP]_0_ = 0.9 M) catalyzed by complexes **9** (curve 5) and **10** (curve 6) at 70 °C.

Hydrogen peroxide is not a good oxidizing agent for alcohols in our case of cobalt catalysts. For example, 1-phenylethanol (0.33 M) after 180 min at 60 °C (other conditions are the same as in Table 1) gave 5.0 mM of acetophenone (yield was only 1.5%) when the reaction was catalyzed by complex **1**. In contrast, *tert*-BuOOH efficiently oxidizes (yield 98% after 13 h) 1-phenylethanol if compound **1** is used as a catalyst (Figure 4).

### 2.6. Stereoselective Oxidation with meta-Chloroperoxybenzoic Acid (MCPBA)

**Figure 5:**
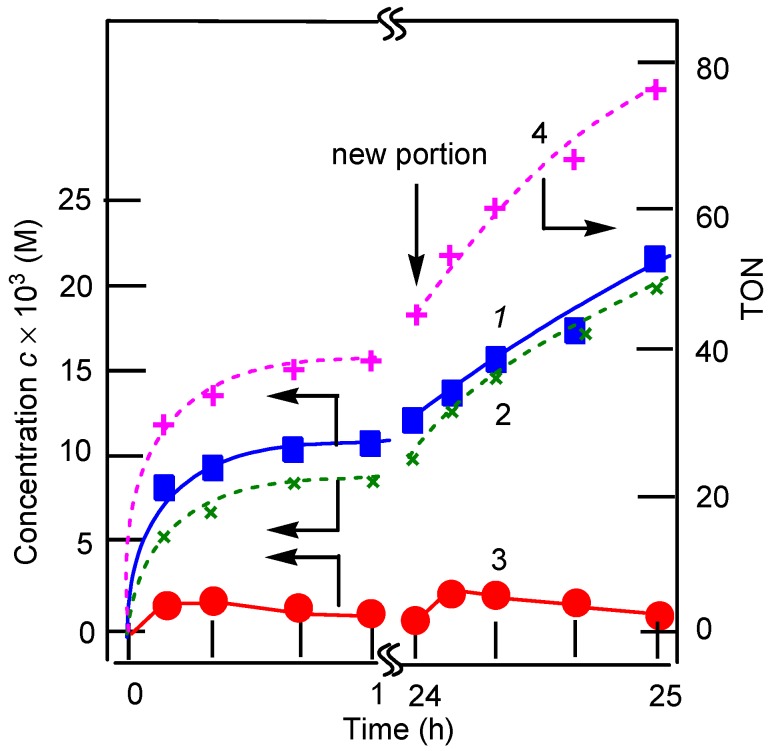
Accumulation of cyclohexanol (curve 1), cyclohexanone (curve 2), and cyclohexyl hydroperoxide (curve 3), as well as TON (curve 4) in the reaction of cyclohexane (0.46 M) with MCPBA (0.025 M) in MeCN at 25 °C catalyzed by Mn(ClO_4_)_2_ (5 × 10^−4^ M). Concentrations of cyclohexanone and cyclohexanol were measured by GC twice: before and after the reduction of the reaction mixture with PPh_3_ [46,47,48,49,50,51,52,53,54,55,56]. At the time moment denoted by an arrow, an additional amount (0.025 M) of MCPBA was introduced into the reaction solution. After 25 h, TON was 76, and the yield of all oxygenates based on MCPBA was 76%.

It turned out that the third oxidant, MCPBA, is a weak oxidant in the reaction with 1-phenylethanol. Thus, the oxidation catalyzed by complex **1** in the presence of HNO_3_ (for the conditions, see Table 1) afforded after 3 h acetophenone in only 18% yield. The oxidation of cyclohexane was also inefficient (Table 2). It should be noted that the chromatogram made before and after reduction of samples with triphenylphosphine as well as the ketone/alcohol ratio is not changed in the chromatograms. This indicates that cyclohexyl hydroperoxide is not formed in the course of the oxidation (for this simple method, see References [46,47,48,49,50,51,52,53,54,55,56]). In contrast, Figure 5, which is presented here for comparison, demonstrates that cyclohexyl hydroperoxide, CyOOH, is produced in the oxidation with MCPBA catalyzed by the salt Mn(ClO_4_)_2_. In the course of the reaction, CyOOH gradually decomposes to afford cyclohexanone and cyclohexanol.

The most impressive results have been obtained in oxidations of alkanes containing tertiary C–H bonds. Thus, cobalt complexes under consideration exhibited relatively high activity in the oxidation of isomers of dimethylcyclohexane with MCPBA (Table 3). The chromatograms of the reaction solutions made before and after reduction of samples with triphenylphosphine are very similar (see Table 3, run 6) and the ketone/alcohol ratio is not changed in the chromatograms. It should be noted that tertiary alkyl hydroperoxides easily decompose in hot injector with splitting C–H bonds and formation of carbonyl derivatives [2]. If tertiary alkyl hydroperoxide is produced the chromatograms before and after reduction with PPh_3_ should be different which is not our case. Thus, alkyl hydroperoxides are not formed in the course of the oxidation (for the method, see References [46,47,48,49,50,51,52,53,54,55,56]). The yield of tertiary alcohols attained 53% based on MCPBA (Table 3, run 22). In many cases shown in Table 3 the reaction proceeds stereoselectively, the highest parameter *trans*/*cis* = 0.05–0.07 was attained for catalysts **1**, **2** and **3**. The lowest ratios *trans*/*cis* have been achieved when nitric acid in low concentration has been added to the reaction solution.

The data collected in Table 3 allow us to compare activities and stereoselectivity parameters of different cobalt complexes. Table 3 indicates sufficiently different catalytic activities and stereoselectivity parameters in the oxidations catalyzed by different cobalt complexes. Addition of nitric acid greatly enhances the efficiency in all cases. In the hydroxylation of *cis*-1,2-dimethylcyclohexane complexes **1** and **8** exhibit the highest activity (yields are 39% and 53% after 2 h). However, the oxidation catalyzed by complex **1** is much more selective (*trans/cis* = 0.05) than that in the presence of compound **8** (*trans/cis* = 0.3). Complexes **9** and **10** containing bulky 1,1′-dipyridil ligands are very poor and non-stereoselective catalysts which is apparently due to sterical hindrance around the reaction center. Previously, Nam et al. [16] in the oxidation catalyzed by Co(ClO_4_)_2_ used large excess of *cis*-1,2-dimethylcyclohexane (1 mmol) over MCPBA (0.02 mmol) and obtained the oxygenates in 80% yield based in the oxidant. We introduced into the reaction low amount of the expensive substrate (140 mM) and larger concentration of MCPBA (260 mM). In our case, hydroxylated products were obtained in high yield: 53% based on the substrate.

### 2.7. Studies of Obtained Complexes by Electrospray Ionization/Mass Spectrometry (ESI-MS) Method

**Figure 6:**
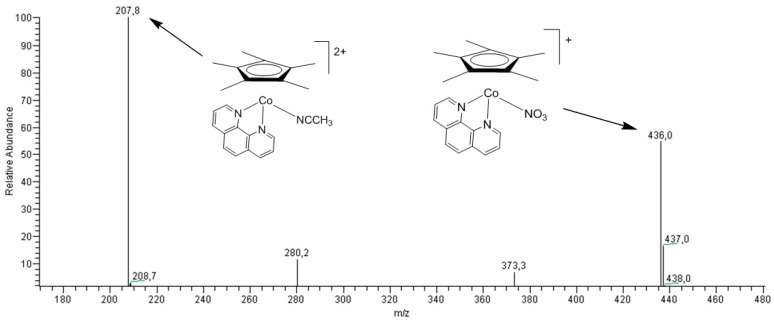
The ESI-MS spectrum (positive-ion mode) of the reaction mixture of complex **7** and HNO_3_ in acetonitrile solution at 55 °C after 1 h. Concentrations: [**7**]_0_ = 6.5 × 10^−4^ M, [HNO_3_]_0_ = 0.04 M. Total volume of the reaction mixture was 3 mL.

In order to determine possible organo-cobalt spices responsible for the catalytic oxidation of alkanes by MCPBA, we investigated the reaction of the obtained cobalt complexes with HNO_3_ by the ESI-MS method, which is a common tool to gain mechanistic insights into homogeneous metal-catalyzed reactions [92]. The primary reaction of containing bidentate nitrogen ligands iodide complexes **7**, **9**, and **10** with HNO_3_ in acetonitrile leads to the replacement of iodide with either a nitrate anion or an acetonitrile molecule. Thus, the ESI-MS spectrum of the reaction mixture with complex **7** (Figure 6) reveals two main peaks corresponding to ionic fragments [Cp*Co(phen)(CH_3_CN)]^2+^ (*m*/*z* = 207.8) and [Cp*Co(phen)(NO_3_)]^+^ (*m*/*z* = 436.0). It can be concluded on the basis of Table 3 (runs 33, 36–39) that complexes **7**, **9**, and **10** containing phenanthroline or dipyridine ligands are the poorest catalysts in the alkane oxygenation.

**Figure 7:**
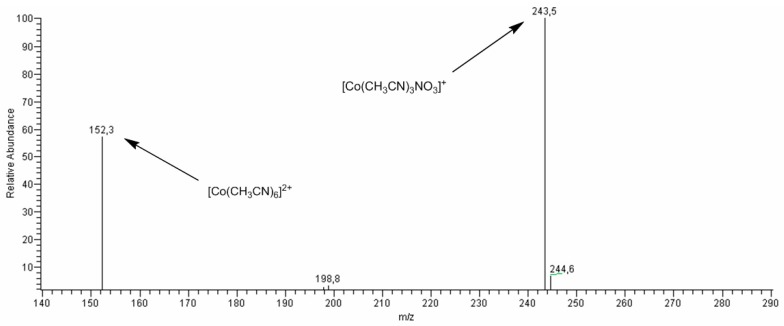
The ESI-MS spectrum (positive-ion mode) of the reaction mixture of complex **6** and HNO_3_ in acetonitrile solution at 55 °C after 1 h. Concentrations: [**6**]_0_ = 6.5 × 10^−4^ M, [HNO_3_]_0_ = 0.04 M. Total volume of the reaction mixture was 3 mL.

In contrast, interaction of HNO_3_ under the same reaction conditions with complexes **3**, **6**, and **8** results in complete decomposition of the complexes to form solvated Co^2+^ salts detected by the appearance of two characteristic peaks in the ESI-MS spectra with *m*/*z* = 152.3 and 243.5 (Figure 7) attributed to species [Co(MeCN)_6_]^2+^ and [Co(MeCN)_3_(NO_3_)]^+^, respectively. The catalysts **3**–**6**, and **8** exhibited in the oxidation moderate yield and stereoselectivity (see Table 3). Noteworthy, although complexes **3**, **6,** and **8** heated with HNO_3_ exhibit the same patterns in the ESI-MS spectra they have different behavior in the catalytic oxidation of *cis*-1,2-DMCH. The oxidation stereoselectivity in the case of complex **8** is lower than that for complexes **3** and **6**. It can be due to lower bulkiness of active species formed from **8** in comparison with the case of pre-catalysts **3** and **6**.

**Figure 8:**
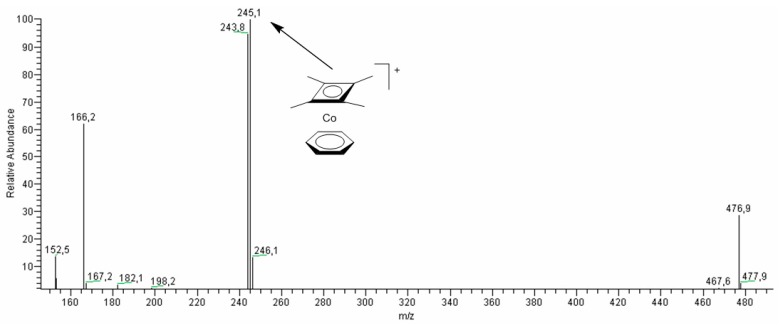
The ESI-MS spectrum (positive-ion mode) of the reaction mixture of complex **1** with HNO_3_ and MCPBA in acetonitrile solution at 55 °C after 1 h. Concentrations: [**1**]_0_ = 6.5 × 10^−4^ M, [HNO_3_]_0_ = 0.04 M, [MCPBA]_0_ = 0.015 M. Total volume of the reaction mixture was 3 mL.

It is interesting that efficient in stereoselective oxidation complex **1** (Table 3, runs 4–19) was significantly more stable toward HNO_3_. After heating in acetonitrile during 1 h at 55 °C the ESI-MS spectrum reveals the sole signal (*m*/*z* = 245) corresponding to the starting cation [Cb*Co(C_6_H_6_)]^+^. However, addition of MCPBA and prolonged heating for 1 h leads to appearance of extra signals in the spectrum (Figure 8), which clearly indicates the interaction of **1** with MCPBA leading to the activation of pre-catalyst [Cb*Co(C_6_H_6_)]^+^.

### 2.8. On the Mechanism of Catalyzed C–H Bond Oxidation with MCPBA

We demonstrated in Section 2.5 that the catalyzed alkane oxidation with H_2_O_2_ affords predominantly alkyl hydroperoxides because the treatment of the reaction solution with PPh_3_ leads to the decrease of the ketone peak and increase of the alcohol peak in GC [55] (compare entries 1 and 2 in Table 1). The cyclohexane oxidation with MCPBA catalyzed by the manganese salt affords some amount of cyclohexyl hydroperoxide (Figure 5). On the contrary, catalysis by cobalt complexes of the oxidation of dimethycyclohexane with MCPBA led to the identical results for the product content measured before and after reduction with PPh_3_ (see Table 3, run 6). Thus, we may conclude that alkyl hydroperoxides are not formed in the oxidation with MCPBA and that free radicals are not involved into the process. The reaction most probably proceeds via the concerted mechanism with peroxide oxygen insertion into the alkane C–H bond [37,42,61].

## 3. Materials and Methods

### 3.1. General

The syntheses were carried out under an inert atmosphere in dry solvents. The isolation of products was conducted in air. Hydrogen peroxide, TBHP, and MCPBA were used as oxidants. Acetonitrile was employed as solvent in all oxidations. ^1^H-NMR spectra (δ in ppm) were recorded on a Bruker Avance-400 spectrometer (Moscow, Russia) (400.13 MHz) relative to residual protons of the solvents.

### 3.2. Syntheses of Complexes

The isolation of products was conducted in air. Catalysts [(C_4_Me_4_)Co(C_6_H_6_)]PF_6_ (**1**) [93], Cp*Co(CO)I_2_ (**2**) [94], CpCo(CO)I_2_ (**3**) [95], (C_4_Me_4_)Co(CO)_2_I (**4**) [93], [(C_4_H_4_BCy)Co(CO)_2_]_2_ (**6**) [96], and [CpCoI_2_]*_n_* (**8**) [97] have been described previously in the literature. Complex [CpCo(bipy)I]PF_6_ (**9**) was prepared by King’s method [95] in high yield, with the use of the improved counterion-exchange procedure described below.

*Compound [(C_4_Me_4_)Co(bipy)(MeCN)]PF_6_* (**5**). A solution of the complex [(C_4_Me_4_)Co(C_6_H_6_)]PF_6_ (**1**) (100 mg, 0.26 mmol) in acetonitrile (3 mL) was irradiated using mercury luminescent lamp (400 W) for 2 h with the use of a running water cooler for 2 h. 2,2′-Bipyridyl (44 mg, 0.28 mmol) was added to the obtained solution. The reaction mixture was stirred overnight. The solvent was removed in vacuo. The residue was extracted with CH_2_Cl_2_ and ether was added. The precipitate was filtered off, washed with ether, and dried in vacuo. Yield of compound [(C_4_Me_4_)Co(bipy)(MeCN)]PF_6_ (**5**) obtained as an orange solid was 63 mg (48%). Anal. Calc. for C_20_H_23_N_3_CoF_6_P:C, 47.16; H, 4.56; N, 8.25%. Found: C, 46.31; H, 4.59; N, 8.52%. ^1^H-NMR (acetone-*d*_6_): δ = 8.99 (d, *J* = 5.2 Hz, 2 H, bipy), 8.66 (d, *J* = 8.0 Hz, 2 H, bipy), 8.29 (m, 2 H, bipy), 7.83 (m, 2 H, bipy), 1.31 (s, 12 H, C_4_Me_4_).

Compound [Cp*Co(phen)I]PF_6_ (**7**). Benzene (5 mL) was added to a mixture of complex Cp*Co(CO)I_2_ (**3**, 120 mg, 0.24 mmol) and phen (56 mg, 0.36 mmol). The reaction mixture was stirred overnight. The solvent was removed in vacuo. The complex [Cp*Co(phen)I]I was extracted from the residue with water. Then an excess of an aqueous KPF_6_ solution was added. The black precipitate that formed was filtered off, washed with water, and dried in vacuo. Reprecipitation from acetonitrile with ether gave complex [Cp*Co(phen)I]PF_6_ (**7**, 70 mg, 45%) as a black solid. Anal. Calc. for C_22_H_23_N_2_CoIF_6_P:C, 40.89; H, 3.59; N, 4.33%. Found: C, 41.03; H, 3.44; N, 4.51%. ^1^H-NMR (acetonitrile-*d*_3_): δ = 9.71 (d, *J* = 5.2 Hz, 2 H, phen), 8.72 (d, *J* = 8.0 Hz, 2 H, phen), 8.14 (m, 4 H, phen), 1.60 (s, 15 H, Cp*).

*Compound [CpCo(bipy)I]PF_6_* (**9**). Complex [CpCo(bipy)I]PF_6_ was prepared by King’s method [95] in high yield, with the use of the improved counterion-exchange procedure given below. Benzene (5 mL) was added to a mixture of complex CpCo(CO)I_2_ [95] (**3**,120 mg, 0.28 mmol) and bipy (73 mg, 0.55 mmol). The reaction mixture was stirred overnight. The precipitate of [CpCo(bipy)I]I was filtered off, washed with benzene and ether, and dried in vacuo. The precipitate was extracted with water. Then an excess of an aqueous KPF_6_ solution was added. The dark violet precipitate that formed was filtered off, washed with water, and dried in vacuo. Reprecipitation from acetone with ether gave complex [CpCo(bipy)I]PF_6_ (**9**, 126 mg, 86%) as a dark violet solid. ^1^H-NMR (acetone-*d*_6_): δ = 10.07 (d, *J* = 5.2 Hz, 2 H, bipy), 8.54 (d, *J* = 8.0 Hz, 2 H, bipy), 8.33 (m, 2 H, bipy), 7.87 (m, 2 H, bipy), 6.18 (s, 5 H, Сp).

Compound [Cp*Co(bipy)I]PF_6_ (**10**). Benzene (5 mL) was added to a mixture of complex Cp*Co(CO)I_2_ (**2**, 120 mg, 0.24 mmol) and bipy (47 mg, 0.36 mmol). The reaction mixture was stirred overnight. The solvent was removed in vacuo. The complex [Cp*Co(bipy)I]I was extracted from the residue with water and methanol. Then an excess of an aqueous KPF_6_ solution was added. The black precipitate that formed was filtered off, washed with water, and dried in vacuo. Reprecipitation from acetone with ether gave complex [Cp*Co(bipy)I]PF_6_ (**10**, 47 mg, 32%) as a black solid. Anal. Calc. for C_20_H_23_N_2_CoIF_6_P:C, 38.62; H, 3.70; N, 4.50%. Found: C, 38.69; H, 3.68; N, 4.46%. ^1^H-NMR (acetone-*d*_6_): δ = 9.61 (d, *J* = 5.6 Hz, 2 H, bipy), 8.58 (d, *J* = 8.0 Hz, 2 H, bipy), 8.35 (m, 2 H, bipy), 7.95 (m, 2 H, bipy), 1.68 (s, 15H, Сp*).

### 3.3. X-ray Diffraction Study

Crystals were grown by slow diffusion in a two-layer system of ether/petroleum ether mixture with solution of complex in acetonitrile (for [Cp*Co(phen)I]PF_6_ (**7**)) or acetone (for [CpCo(bipy)I]PF_6_ (**9**) and [Cp*Co(bipy)I]PF_6_ (**10**)). X-ray diffraction data for **10** were collected with Bruker Apex2 DUO diffractometer (Moscow, Russia) at 100 K and those for **7** and **9** with Bruker Apex 2 diffractometer at 120 and 100 K, respectively, using graphite monochromated Mo-Kα radiation (λ = 0.71073 Å, ω-scans). The structures were solved by the direct method and refined by the full-matrix least-squares against F^2^ in anisotropic approximation for non-hydrogen atoms. The positions of hydrogen atoms were calculated, and they were refined in isotropic approximation in riding model. Crystal data and structure refinement parameters for **7**, **9**, and **10** are collected in Table 4. All calculations were performed using the SHELXTL PLUS 5.0 [98]. CCDC 1504665, 1504667 and 1504666 contain the supplementary crystallographic data for **7**, **9**, and **10**, respectively. These data can be obtained free of charge via www.ccdc.cam.ac.uk/data_request/cif.

The molecular structures, bond lengths and angles as well as crystallographic data and structure refinement parameters of the three catalysts **7**, **9**, and **10** obtained in this work are given in Figure 1, Figure 2 and Figure 3 above and Table 4 below.

### 3.4. Catalytic Oxidation of Alkanes and 1-Phenylethanol

Typically, catalyst and the co-catalyst (nitric or trifluoroacetic acid) were introduced into the reaction mixture in the form of stock solutions in acetonitrile. The reactions of alcohols and hydrocarbons were carried out in air in thermostated Pyrex cylindrical vessels with vigorous stirring and using MeCN as solvent. The substrate (alcohol or hydrocarbon) was then added and the reaction started when the oxidant was introduced in one portion. (CAUTION: The combination of air or molecular oxygen and peroxides with organic compounds at elevated temperatures may be explosive!). The reactions with 1-phenylethanol were analyzed by ^1^H-NMR method (solutions in acetone-*d*_6_; “Bruker AMX-400” instrument, 400 MHz). Areas of methyl group signals were measured to quantify oxygenates formed in oxidations of 1-phenylethanol. As we made previously, the samples obtained in the alkane oxidation were typically analyzed twice (before and after their treatment with PPh_3_) by GC. This method (comparison of chromatograms of the same sample obtained before and after addition of PPh_3_) was proposed by Shul’pin earlier [46,47,48,49,50,51,52,53,54,55,56] and allows us to estimate real concentration of alkyl hydroperoxide, ketone (aldehyde and alcohol) present in the reaction solution. Samples of the reaction mixture were analyzed by GC (the chromatograph-3700; fused silica capillary column FFAP/OV-101 20/80 *w*/*w*, 30 m × 0.2 μm × 0.3 μm; helium as a carrier gas). Attribution of peaks was made by comparison with chromatograms of authentic samples and by GC–MS (INEOS, Moscow, Russia).

## Figures and Tables

**Table 1:** Oxidation of cyclohexane with hydrogen peroxide catalyzed by cobalt complexes ^1^.

Entry	Cat	Time (min)	Reduction with PPh_3_	Cyclohexanone (mM)	Cyclohexanol (mM)	TON	TOF (h^‒1^)
1	**1**	30	No	0.05	0.07	0.24	0.48
2		30	Yes	0	0.3	0.6	1.2
3		120	No	0.05	0.08		
4		120	Yes	0	0.7		
5	**5** ^2^	60	Yes	0.1	0.6		
6		180	Yes	0.5	1.0		
7	**7**	120	Yes	0	1.4		
8	**7** ^2^	60	Yes	0.2	1.0		
9		120	Yes	1.0	2.3	6.6	3.3

^1^ Conditions. Concentrations [**Cat**]_0_ = 5 × 10^−4^ M, [cyclohexane]_0_ = 0.46 M, [H_2_O_2_]_0_ = 1.5 M, 50 °C. Solvent MeCN, total volume of the reaction solution was 5 mL; ^2^ Nitric acid (0.05 M) was added.

**Table 2:** Oxidation of cyclohexane with *meta*-chloroperoxybeanzoic acid (MCPBA) catalyzed by cobalt complexes ^1^.

Entry	Cat	Time (min)	HNO_3_ (mM)	Cyclohexanone (mM)	Cyclohexanol (mM)	Total Yield (%)
1	**1**	120	50	18	5	16
2	**5**	120	0	26	6	23
3		120	50	20	4.5	18
4	**6**	60	50	0.7	2.6	3
5		180	50	1.9	5.3	5

^1^ Conditions. Concentrations [**Cat**]_0_ = 5 × 10^−4^ M, [cyclohexane]_0_ = 0.23 M, [MCPBA]_0_ = 0.26 M. Solvent MeCN, total volume of the reaction solution was 5 mL. For entry 2, TON = 106, TOF=53 h^‒1^.

**Table 3:** Oxygenation of isomeric dimethylcyclohexanes with MCPBA catalyzed by cobalt complexes ^1^.

Run	Cat	Substrate	[HNO_3_] (mM)	Time (min)	*t*-Dimethyl-Alcohol (mM)	*c*-Dimethyl-Alcohol (mM)	Yield (%)	Ratio *trans*/*cis*
1	None	*cis*-1,2-DMCH	0	30	0.13	0.17	0.2	0.76
2	None	*cis*-1,2-DMCH	0	120	2.9	4.1	5	0.74
3	None	*cis*-1,2-DMCH	50	30	0.07	0.5	0.4	0.14
4	**1**	*cis*-1,2-DMCH	0	30	3	23	19	0.13
5		*cis*-1,2-DMCH	0	120	4	29	24	0.14
6		*cis*-1,2-DMCH ^2^	50	120	2.4	52	39	0.05
7		*cis*-1,2-DMCH	50 ^3^	30	3	11	10	0.30
8		*cis*-1,2-DMCH	50 ^3^	120	5	19	17	0.26
9		*cis*-1,2-DMCH	50 ^4^	30	8	12	14	0.70
10		*trans*-1,2-DMCH	0	30	19	8	19	2.4
11		*trans*-1,2-DMCH	0	120	19	8	19	2.4
12		*trans*-1,2-DMCH	50	30	26	1	19	19
13		*trans*-1,2-DMCH	50	120	36	2	27	21
14		*trans*-1,2-DMCH	50	30	13	8	15	1.6
15		*trans*-1,2-DMCH	50 ^4^	120	14	9	16	1.5
16		*cis*-1,4-DMCH	50	30	3	48	37	0.07
17		*cis*-1,4-DMCH	50	120	14	9	43	0.11
18		*trans*-1,4-DMCH	50	30	30	3	23	12
19		*trans*-1,4-DMCH	50	120	30	2	23	18
20	**2**	*cis*-1,2-DMCH	0	120	10	32	30	0.28
21		*cis*-1,2-DMCH	50	30	3.5	44	34	0.08
22		*cis*-1,2-DMCH	50	120	4	69	53	0.06
23	**3**	*cis*-1,2-DMCH	50	30	3.5	50	35	0.07
24		*cis*-1,2-DMCH	50	120	4	61	46	0.07
25	**4**	*cis*-1,2-DMCH	50	30	3	37	26	0.08
26		*cis*-1,2-DMCH	50	120	3	44	32	0.07
27		*trans*-1,4-DMCH	50	30	24	1	17	27
28		*trans*-1,4-DMCH	50	120	35	1	26	34
29	**5**	*cis*-1,2-DMCH	50	90	9	14	16	0.64
30		*cis*-1,2-DMCH	50	180	4	10	10	0.4
31	**6**	*cis*-1,2-DMCH	50	30	3	39	30	0.08
32		*cis*-1,2-DMCH	50	120	3	40	30	0.07
33	**7**	*cis*-1,2-DMCH	50	300	3	6	6	0.5
34	**8**	*cis*-1,2-DMCH	50	30	17	54	50	0.3
35		*cis*-1,2-DMCH	50	120	17	57	53	0.3
36	**9**	*cis*-1,2-DMCH	50	30	4.5	6	7	0.82
37		*cis*-1,2-DMCH	50	120	4.5	6	7	0.82
38	**10**	*cis*-1,2-DMCH	50	30	3	4	5	0.84
39		*cis*-1,2-DMCH	50	120	3	4	5	0.84

^1^ Reactions were performed in MeCN (total volume 2.5 mL). Concentrations: [Substrate]_0_ = 140 mM, [MCPBA]_0_ = 260 mM, catalyst [**Cat**]_0_ = 0.5 mM, 40 °C. Samples were analyzed by GC after reduction with PPh_3_. *t*-Dimethyl and *c*-dimethyl alcohol are tertiary alcohols with *trans*- and *cis*-mutual orientation of methyl groups, respectively. The ratio *trans*/*cis* is the ratio of these two isomers. Parameters for entries 22 and 35 were TON = 150 and TOF = 75 h^‒1^; ^2^ The chromatogram obtained for the reaction solution before reduction with PPh_3_ (*t*-dimethyl alcohol, 2.5 mM; *t*-dimethyl alcohol, 52 mM) did not practically distinguish from the data obtained for the reduced sample; ^3^ Trifluoroacetic acid (50 mM) was used instead of nitric acid; ^4^ Salt LiNO_3_ (50 mM) was used instead of nitric acid.

**Table 4:** Crystallographic data and structure refinement parameters for **7**, **9**, and **10**.

Compound	7	9	10
Empirical formula	C_22_H_23_CoF_6_IN_2_P	C_15_H_13_CoF_6_IN_2_P	C_20_H_23_CoF_6_IN_2_P
Formula weight	646.22	552.07	622.20
Crystal system	Triclinic	Orthorhombic	Monoclinic
Space group	*P-1*	*Pna2_1_*	*P2_1_/n*
*a* (Å)	8.2684(9)	16.7546(14)	9.0966(18)
*b* (Å)	11.4505(13)	8.4636(7)	26.448(5)
*c* (Å)	12.4520(14)	24.681(2)	9.2018(17)
α (°)	87.800(2)	-	-
β (°)	84.628(2)	-	90.574(4)
γ (°)	77.714(2)	-	-
*V* (Å^3^)	1146.7(2)	3499.9(5)	2213.7(7)
*D*_calc_ (g·cm^−^^3^)	1.872	2.095	1.867
Linear absorption, *μ* (cm^−^^3^)	22.27	28.98	23.02
F(000)	636	2128	1224
2θ_max_, °	56	52	54
Reflections measured	20,367	31,839	28,418
Independent reflections	5529 (*R*_int_ = 0.0416)	6888 (*R*_int_ = 0.0642)	4819 (*R*_int_ = 0.1797)
Observed reflections (*I* > 2σ(*I*))	4730	6398	4320
Parameters	303	484	307
*R_1_*	0.0301	0.0620	0.0739
*wR_2_*	0.0800	0.1721	0.2145
Goodness-of-fit	1.017	1.271	1.477
Largest diff. peak and hole (e Å^−^^3^)	1.44 and −0.72	2.49 and −1.51	2.34 and −1.92
